# Metabolic profiles of meconium in preeclamptic and normotensive pregnancies

**DOI:** 10.1007/s11306-025-02224-4

**Published:** 2025-01-25

**Authors:** Elli Toivonen, Jutta Sikkinen, Anne Salonen, Olli Kärkkäinen, Ville Koistinen, Anton Klåvus, Topi Meuronen, Tuomas Heini, Arina Maltseva, Mikael Niku, Tiina Jääskeläinen, Hannele Laivuori

**Affiliations:** 1 Center for Child, Adolescent and Maternal Health Research, Faculty of Medicine and Health Technology, Tampere University, Tampere, Finland; 2 Department of Obstetrics and Gynecology, Tampere University Hospital, The Wellbeing Services County of Pirkanmaa, Tampere, Finland; 3 Human Microbiome Research Program, Faculty of Medicine, University of Helsinki, Helsinki, Finland; 4 Afekta Technologies Ltd., Kuopio, Finland; 5 Department of Veterinary Biosciences, University of Helsinki, Helsinki, Finland; 6 Medical and Clinical Genetics, University of Helsinki and Helsinki University Hospital, Helsinki, Finland; 7 Department of Food and Nutrition, University of Helsinki, Helsinki, Finland; 8 Institute for Molecular Medicine Finland, Helsinki Institute of Life Science, Helsinki, Finland; 9 School of Pharmacy, University of Eastern Finland, Kuopio, Finland

**Keywords:** Preeclampsia, Meconium, Metabolome, Metabolomics, Pregnancy

## Abstract

**Introduction:**

Preeclampsia (PE) is a common vascular pregnancy disorder affecting maternal and fetal metabolism with severe immediate and long-term consequences in mothers and infants. During pregnancy, metabolites in the maternal circulation pass through the placenta to the fetus. Meconium, a first stool of the neonate, offers a view to maternal and fetoplacental unit metabolism and could add to knowledge on the effects of PE on the fetus and newborn.

**Objectives:**

To compare meconium metabolome of infants from PE and normotensive pregnancies.

**Methods:**

A cohort of preeclamptic parturients and normotensive controls were recruited in Tampere University Hospital during 2019–2022. Meconium was sampled and its metabolome analyzed using liquid chromatography– mass spectrometry in 48 subjects in each group.

**Results:**

Differences in abundances of 1263 compounds, of which 19 could be annotated, were detected between the two groups. Several acylcarnitines, androsterone sulfate, three bile acids, amino acid derivatives (phenylacetylglutamine, epsilon-(gamma-glutamyl)lysine and N-(phenylacetyl)glutamic acid), as well as caffeine and paraxanthine were lower in the PE group compared to the control group. Urea and progesterone were higher in the PE group.

**Conclusion:**

PE is associated with alterations in the meconium metabolome of infants. The differing abundances of several metabolites show alterations in the interaction between the fetoplacental unit and mother in PE, but whether they are a cause or an effect of the disorder remains to be further investigated.

**Supplementary Information:**

The online version contains supplementary material available at 10.1007/s11306-025-02224-4.

## Introduction

Preeclampsia (PE) is a potentially fatal disorder that complicates several million pregnancies annually, with regionally varying incidence of 1-5.6% (Dimitriadis et al., 2023). It is a systemic vascular disorder with alterations in physiologic metabolic changes during pregnancy (Chiarello et al., 2020). The impact of PE extends beyond pregnancy, as numerous studies have demonstrated increased morbidity, mainly cardiovascular, in PE survivors and their offspring. However, the mechanisms and causations of related disorders are poorly understood (Burton et al., 2016; Markopoulou et al., 2019; Sehgal et al., 2023).

Metabolite profiles have been suggested to be useful in early diagnosis of diseases (Kenny et al., 2010; Romero et al., 2010) and in understanding the mechanistic pathways of complex disorders. During pregnancy, metabolites in the maternal circulation pass through the placenta to the fetus, including metabolites produced or modulated by the maternal microbiota (Gomez De Agüero et al., 2016; Husso et al., 2023). Meconium, the first stool of the neonate, forms during the last two trimesters of pregnancy and contains molecules transferred through the placenta as well as those resulting from fetal metabolism (Bekhti et al., 2022). PE is associated with impaired placentation and placental function (Steegers et al., 2010), and alterations in the maternal as well as newborn plasma metabolome have been reported in preeclamptic pregnancies (Illsinger et al., 2010; Jääskeläinen et al., 2018, 2021). However no studies describing the meconium metabolites in PE pregnancies have been published. As meconium offers a view to both maternal and fetoplacental unit metabolism, studying the meconium metabolome could add to understanding the inadequately known mechanisms of the effects of PE on the offspring.

Relatively few reports on meconium metabolome measured with non-targeted metabolomics approach have been published, and they concentrate on the change in meconium composition during the first days of life. Proton nuclear magnetic resonance analysis has been used to observe evolution of meconium composition between the first and third day of life, showing mainly increasing concentrations in both water-soluble and organic fractions (Righetti et al., 2003). Similarly, a study using liquid chromatography– mass spectrometry (LC-MS) reported differences between samples that were excreted within 24 h of birth and samples that were excreted later (Bekhti et al., 2022). Even more recently, high-resolution nuclear magnetic resonance spectra of meconium and stools of three pairs of premature twins showed differences of metabolomes according to newborn sex and time of sampling (Trimigno et al., 2024).

In this study we compared the metabolome of meconium of infants from PE and normotensive pregnancies.

## Methods

### Study population and samples

PremiuM (Pre-eclamptic microbiota and metabolism) is a cohort including 51 women with PE and 53 control women who have given birth in Tampere University Hospital during 2019–2022. Women were recruited in late pregnancy or immediately before delivery. At least 18 years old Caucasian women with a singleton pregnancy and gestational age ≥ 28^0/7^ weeks were eligible for the study. To control confounding, women with inflammatory bowel disease, systemic or vaginal antibiotic treatment within three months of delivery, pregestational diabetes or medically treated gestational diabetes were excluded, as well as those who smoked during pregnancy. PE was defined using International Society for the Study of Hypertension in Pregnancy criteria (Brown et al., 2018): gestational hypertension accompanied by proteinuria, other maternal organ dysfunction, or uteroplacental dysfunction. Further exclusion criteria in the control group were hypertensive pregnancy disorders and fetal growth restriction.

The PremiuM study protocol was approved by the Ethics Committee of Pirkanmaa Hospital District (R18052). All participants provided written informed consent.

Meconium was sampled from a diaper using a sterile blade as soon as possible after its first appearance (median 11 h of age, interquartile range 6.9–16.6 h of age). Samples were frozen and stored at -70˚C. Meconium samples were available from 48 PE and 48 control pregnancies.

During analysis, the samples were homogenized by adding cold 80% v/v aqueous LC-MS ultra-grade methanol in a ratio of 1500 µL per 100 mg of sample for the metabolite extraction and protein precipitation using Bead Ruptor 24 Elite homogenizer at the speed 6 m/s at 2 ± 2 °C for 30 s. The samples were incubated on ice for 15 min and vortexed for 10 s followed by centrifugation for 10 min at 4 °C and 17,000 × *g*. The supernatant was collected and filtered (Captiva ND filter plate 0.2 μm) by centrifuging for 5 min at 700 × *g* at 4 °C and kept at 4 °C until analysis. Aliquots of 40 µL were taken from each sample, mixed in one tube, and filtered as reported above to be used as the quality control samples in the analysis.

### Liquid chromatography– mass spectrometry analysis

The samples were analyzed by LC-MS on Agilent 6546 Q-TOF LC-MS System with Agilent Jet Stream source and 1290 Infinity II UHPLC system. The analytical method has been described in more detail in previous reports (Hanhineva et al., 2015; Klåvus et al., 2020). In brief, a Zorbax Eclipse XDB-C18 column (2.1 × 100 mm, 1.8 μm; Agilent Technologies) was used for the reversed-phase (RP) separation and an Acquity UPLC BEH amide column (Waters) for the HILIC separation. After each chromatographic run, the ionization was carried out using jet stream electrospray ionization (ESI) in the positive and negative modes, yielding four data files per sample. The collision energies for the MS/MS analysis were selected as 10, 20, and 40 V, for compatibility with spectral databases.

The mass spectrometric peaks of LC-MS were collected and aligned across samples using MS-DIAL ver. 4.90 (Tsugawa et al., 2015). Data preprocessing and cleanup was performed with the *notame* R package as described by Klåvus et al., 2020. Briefly, the data were corrected for analytical drift, and low-quality molecular features were flagged. Low-quality features were not included in multiple testing correction. Missing values were imputed using random forest imputation for high-quality features and zero imputation for low-quality features. Differential metabolites were identified by comparing their measured properties with in-house and publicly available library entries. The annotation of each metabolite and the level of identification was given based on the recommendations published by the Chemical Analysis Working Group (CAWG) Metabolomics Standard Initiative (MSI) (Sumner et al., 2007). Supplementary material S1 describes the methodology in more detail.

Based on inspection of the distribution of molecular features in each sample and dimensionality reduction visualization, it was noted that two samples (one in the two RP modes and one in all modes) presented very low intensities of feature abundances, hinting to errors in the analysis of the samples. Including these samples in statistical analyses would skew the results, thus they were excluded in statistical analyses of erratic modes. The number of samples per group included in feature-wise analysis per analytical mode: HILIC positive and negative, 48 PE and 48 control group samples; RP positive and negative, 47 PE and 48 control group samples. For multivariate analysis, only samples with complete observations in all modes were used (47 PE and 48 control group samples).

To annotate more features and to explore their potential microbial origin, the final set of molecular features was aligned to a previously published set of fetal metabolites modulated by maternal microbiota (Pessa-Morikawa et al., 2022), based on MS/MS (MS2 tolerance 0.025 Da), retention time (+-0.3’), and m/z (+-0.01 Da), using MS-DIAL ver. 5.2.

### Bacterial PCR analyses

A nested quantitative PCR approach was used to evaluate the bacterial positivity of the meconium samples using primers targeting the 16 S rRNA gene present in all bacteria. PCRs were run with negative DNA extraction and PCR controls, and with genomic DNA from *Bifidobacterium longum* as a positive control as described in Supplementary material S1. Samples were considered positive when their quantification cycle (Cq) value was lower than that of negative controls on the same plate, and melting curve analysis indicated a specific product similar to the positive control. If only one of these criteria were met the samples were coded inconclusive, and samples with the same or higher Cq than negative controls were coded as negative.

### Statistical analyses and data visualization

Statistical analyses were performed using IBM SPSS for Windows version 29 (IBM Corp, Armonk, NY, USA) and R software version 4.2.1 (R Core Team, 2022), and the latter was used for data visualization. Demographic data as well as data on deliveries and newborns of women with PE were compared to that of control women. These data are expressed as medians and minimum and maximum values or as percentages. Chi-square test, Fisher’s exact test and Mann-Whitney U-test were used as appropriate.

Abundances of molecular features identified by LC-MS were compared using linear models with ranks of molecular feature abundances as the dependent variable and study group as the independent variable (which corresponds to a Mann-Whitney U-test between the two study groups). We also adjusted the model for covariates using gestational age, antibiotic use and presence of infection, delay from birth to sample collection, or, in a separate analysis, the presence of bacterial DNA as the covariate. We conducted a sensitivity analysis to control confounding by prematurity by excluding seven study subjects with deliveries before < 34 gestational weeks.

P-values from the tests were adjusted for multiple comparisons using the Benjamini–Hochberg procedure for controlling the false discovery rate (FDR). Adjusted p-values are referred to as q-values. Effect sizes were described using fold changes and Cohen’s d.

In all analyses, a q-value < 0.05 was considered statistically significant and a p-value < 0.05 as nominally significant.

## Results

Women in the PE group were more often primiparous and had higher blood pressure in the first antenatal visit compared to the control group. No differences were observed in maternal age, body mass index, use of assisted reproductive technologies, dietary habits or smoking between the groups, neither regarding incidence of gestational diabetes, mode of delivery or use of antibiotics during labor. Infants were smaller, more often small for gestational age (SGA, birth weight < 2SD) and born at earlier gestation in the PE group compared to the control group. See details in Table 1.

**Table 1:** Data on parturients, pregnancies and newborns

		Preeclampsia *n* = 48			Normotensive *n* = 48		*p* value
		median or n	min, max or %		median or n	min, max or %	
**Maternal characteristics**							
Maternal age, years		30.0	19, 46		30.0	23, 42	0.823
Primiparous		37	77.1		24	50.0	0.006
Body mass index, kg/m^2^		25.4	17.5, 39.9		24.5	17.5, 36.7	0.476
Spontaneuos pregnancy		39	81.3		42	87.5	0.399
IVF or ICSI		7	14.6		4	8.3	0.537
Regular nicotine product use before pregnancy^a^ - Missing data		5 4	11.4		4 8	10.0	0.840
Mixed diet^a^ - Missing data		38 5	88.4		37 10	97.4	0.123
Probiotic use at least weekly^a^ - Missing data		6 6	14.3		8 11	21.6	0.394
ASA (PE prophylaxis)		6	13.3		2	4.2	0.150*
Highest antenatal systolic blood pressure, mmHg		158	133, 209		124	95, 151	< 0.001
Highest antenatal diastolic blood pressure, mmHg		103.5	90, 119		73.5	49, 89	< 0.001
Proteinuria		43	89.6		0		< 0.001
Gestational diabetes		15	31.3		16	34.0	0.772
Vaginal delivery		25	52.1		29	60.4	0.411
Corticosteroid prophylaxis		17	35.4		4	8.3	0.001
Antibiotic prophylaxis for GBS		7	14.6		7	14.6	1
Delay in meconium sampling (min) - Delay ≥ 24 h		753 5	92, 4763 10.6		657 2	10, 1650 4.3	0.452 0.227
qPCR status - positive - negative - inconclusive		19 15 10	43.2 34.1 22.7		21 11 15	44.7 23.4 31.9	0.445
**Fetal characteristics**							
Birth weight, g		2950	1016, 4356		3750	2085, 4935	< 0.001
Birth weight SD		-0.4	-3.1, 1.9		0.5	-1.3, 3.2	< 0.001
SGA (≤ -2.0 SD)		5	10.4		0		0.022
LGA (≥ 2.0 SD)		0			4	8.3	0.041
GA at delivery, weeks		37.4	28.7, 41.9		39.4	34.4, 42.0	< 0.001
GA at delivery < 34 weeks		8	16.7		0		0.003*
Male sex		26	54.2		27	56.3	0.837
Apgar < 7 at age of 5 min		6	12.8		0		0.011
Umbilical artery pH - Missing data		7.28 5	6.94, 7.37		7.26 1	7.00, 7.46	0.340

^a^self-reported *Fisher’s exact

IVF, In vitro fertilization; ICSI, Intracytoplasmic sperm injection; ASA, Acetosalicylic acid; PE, Preeclampsia; GBS, Group B streptococcus; qPCR, Quantitative polymerase chain reaction; SD, Standard deviation; SGA, Small for gestational age; LGA, Large for gestational age; GA, Gestational age

**Fig. 1:**
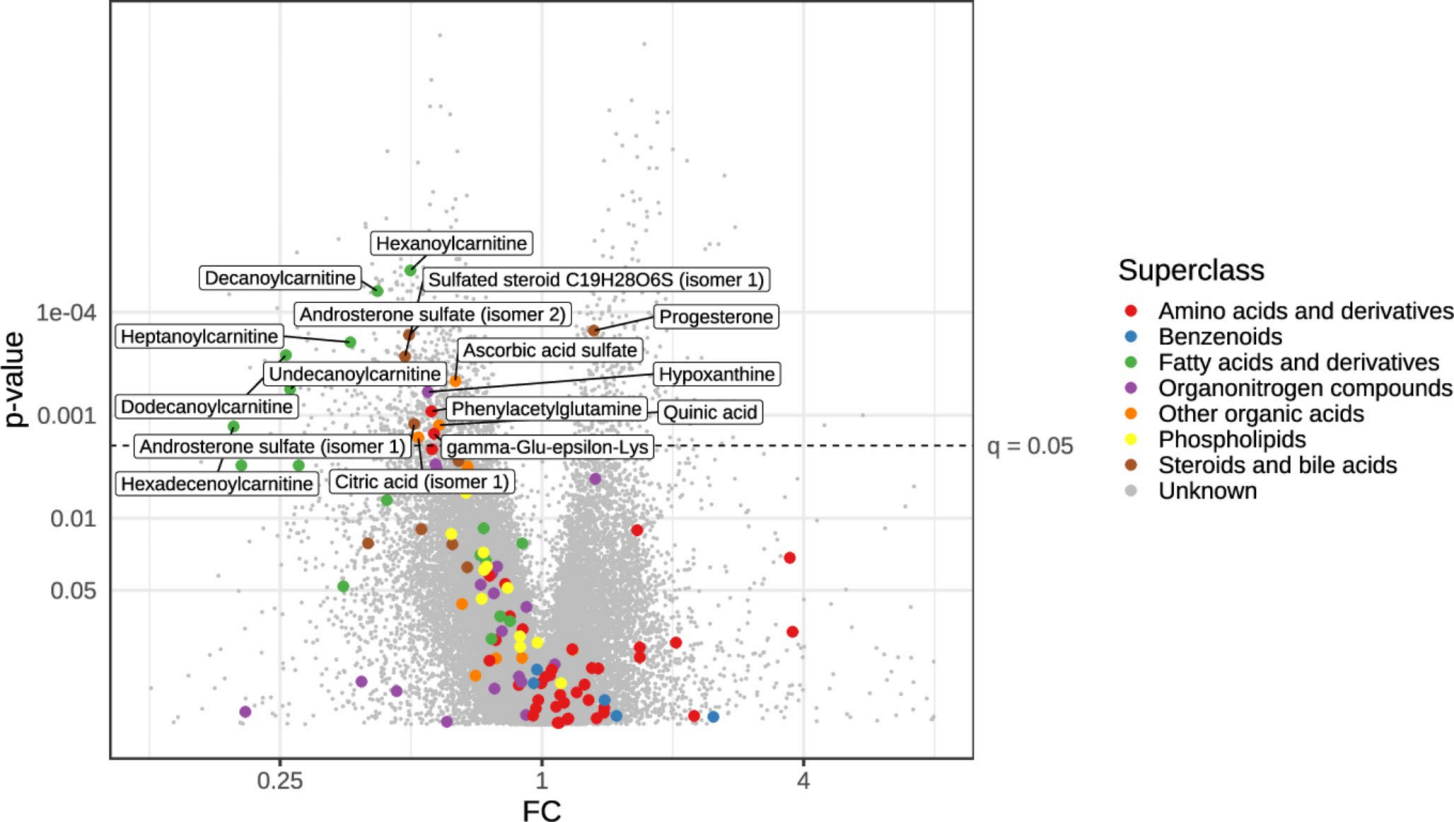
Volcano plot of high-quality features with color denoting metabolite group of annotated compounds. Metabolites with lower and higher abundances in the PE group compared to the control group are located to the left and to the right of zero on the X axis, respectively

**Fig. 2:**
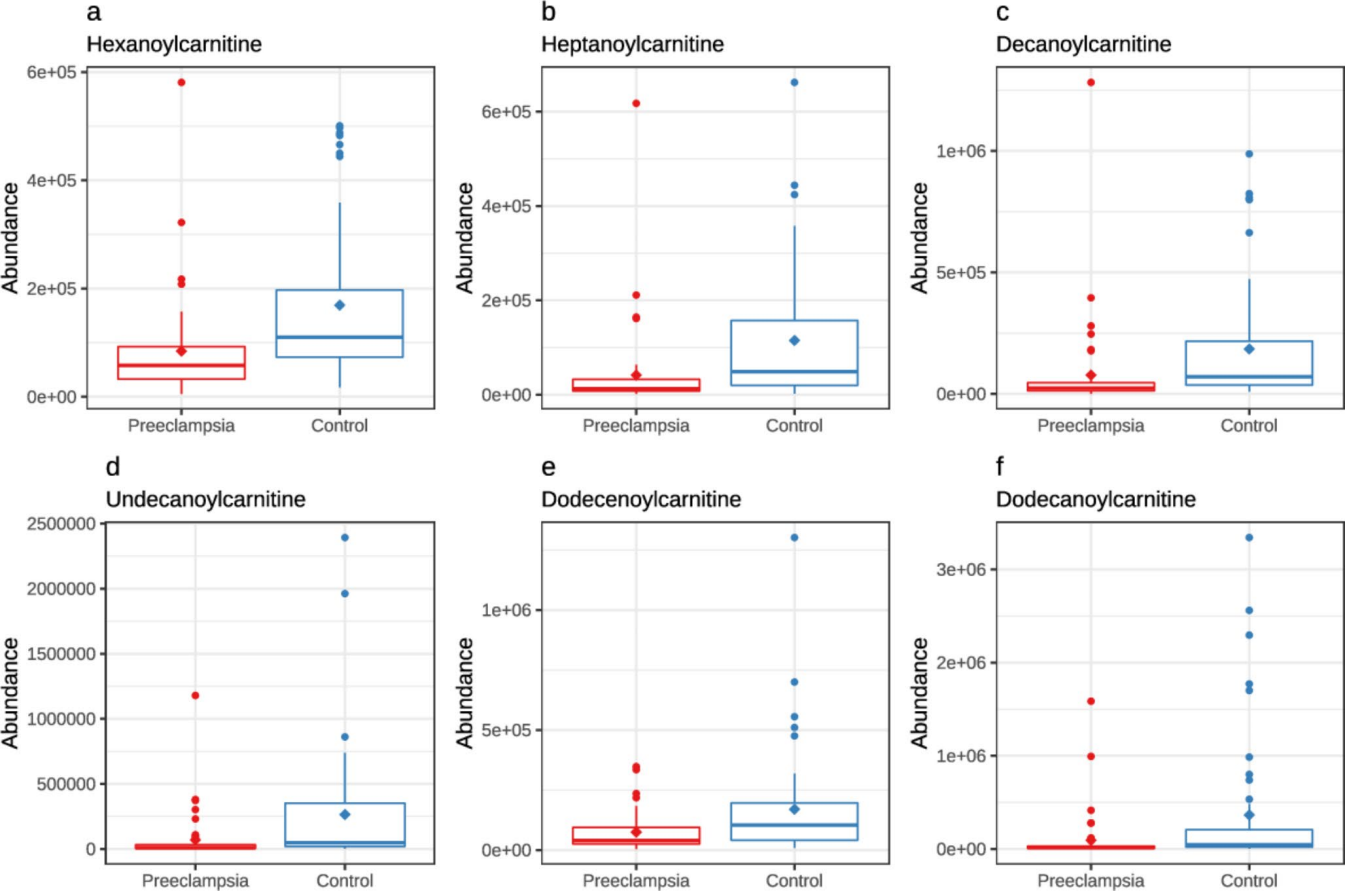
Abundances of differentially abundant acylcarnitines in preeclamptic and normotensive women

There were 31 773 high quality molecular features measured from the meconium samples, 1263 differed statistically significantly between the study groups, and of these differing features, a total of 19 compounds could be annotated. Figure 1 shows a volcano plot of all differing metabolites, detailed in Supplementary Table S2. The most noteworthy differences between the study groups were abundances of several acylcarnitines, which were significantly lower in samples of the PE group than of the control group. These included hexanoylcarnitine (CAR 6:0; *p* <.001 and q = 0.013), heptanoylcarnitine (CAR 7:0; *p* <.001 and q = 0.021), decanoylcarnitine (CAR 10:0; *p* <.001 and q = 0.013), undecanoylcarnitine (CAR 11:0; *p* <.001 and q = 0.031), dodecanoylcarnitine (CAR 12:0; *p* <.001 and q = 0.023), and hexadecenoylcarnitine (CAR 16:0; *p* =.001 and q = 0.042; Fig. 2).

Regarding amino acids and their derivatives, phenylacetylglutamine (*p* <.001, q = 0.037) and epsilon-(gamma-glutamyl)lysine (*p* <.001, q = 0.03) were significantly lower and N-acetylhistidine (*p* =.002, q = 0.051) and histidine betaine (*p* =.004, q = 0.066) nominally lower in the PE group compared to the control group.

**Fig. 3:**
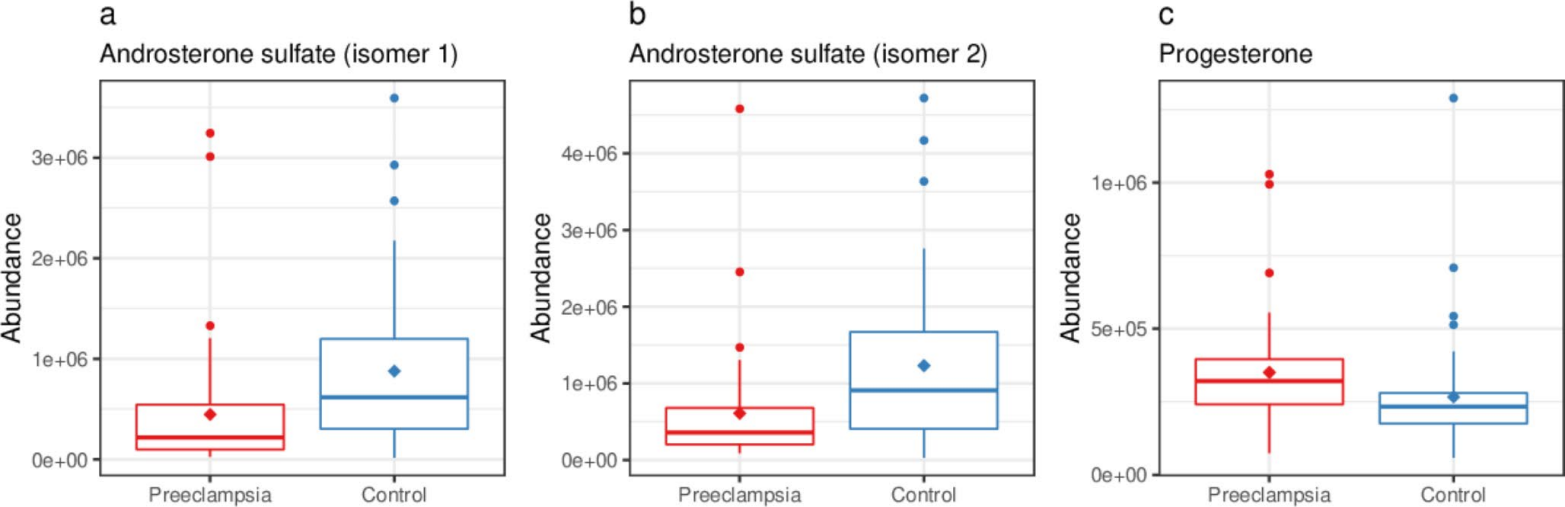
Abundances of two androsterone sulfate isomers and progesterone in preeclamptic compared to normotensive women

Androsterone sulfate (two isomers, *p* <.001, q = 0.019 and *p* =.0012, q = 0.041) was significantly lower and and sulfocholic acid (*p* =.0028, q = 0.056) nominally lower in the PE group compared to the control group. Glycocholic acid (*p* =.018, q = 0.12) and taurocholic acid (*p* =.017, q = 0.012) were nominally lower and progesterone significantly higher (*p* <.001, q = 0.019) in the PE group compared to the control group. The abundance of urea tended to be higher in the PE group than in the control group (*p* =.0024, q = 0.053). Abundances of androsterone sulfate and progesterone are shown in Fig. 3.

**Fig. 4:**
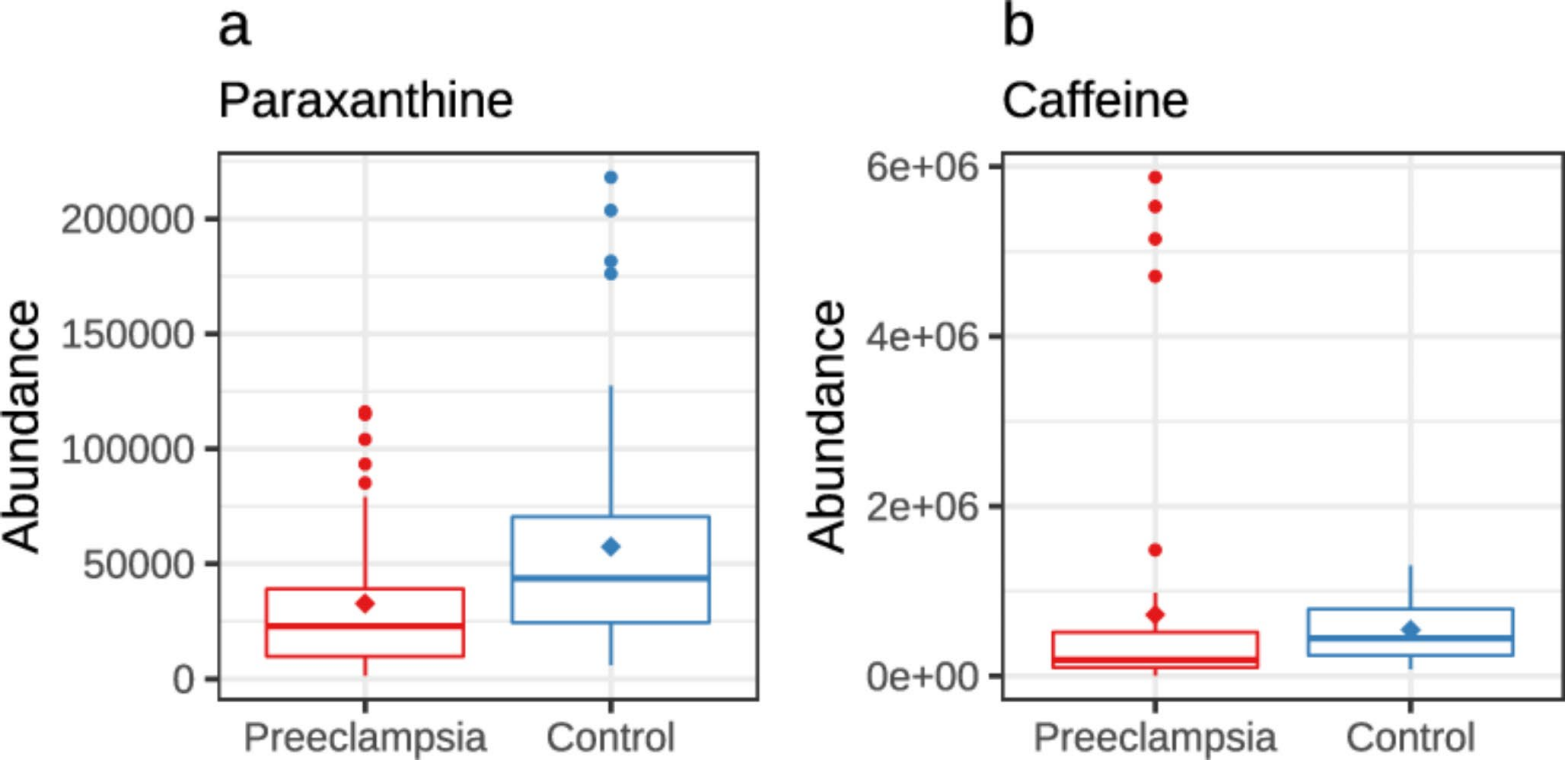
Abundances of caffeine and paraxanthine in preeclamptic and normotensive women

The abundance of caffeine was nominally higher in the PE group meconium samples (*p* =.004, q = 0.065). However, sensitivity analysis showed four outliers, all born < 34 gestational weeks, and after excluding these subjects the abundance of caffeine was lower in the PE group. Abundance of caffeine metabolite paraxanthine was nominally lower in the PE group (*p* =.003, q = 0.057; Fig. 4). Abundances of quinic acid (*p* =.001, q = 0.04) and citric acid (*p* =.002, q = 0.005) were significantly lower in the PE group.

Eleven of the significantly differing molecular features matched the dataset of maternal microbiota-related metabolites (Pessa-Morikawa et al., 2022), and six of the differing annotated metabolites are linked to microbiota in literature. These potentially microbially modulated metabolites included N-(phenylacetyl)glutamic acid (Pessa-Morikawa et al., 2022), phenylacetylglutamine (Krishnamoorthy et al., 2024), hypoxanthine (Xiao et al., 2021), quinic acid (Couteau et al., 2001) and the three bile acids: sulfocholic acid, glycocholic acid and taurocholic acid (Muller et al., 2021; Zarei et al., 2022).

Main results were adjusted by the presence of intrapartum infection, administration of antibiotics, being SGA, and time interval between delivery and meconium sampling. These adjustments had no significant effect on the difference between study groups.

To further control confounding, the effects of traits that differed between the groups as well as characteristics suggested to affect meconium metabolome were assessed. Primiparity, low-dose aspirin use, gestational diabetes or intrapartum infection had no effects on metabolites. When the groupwise differences were adjusted for gestational age, the only metabolite with q < 0.05 was 1-methyl-3-phenylpropylamine, a metabolite of labetalol, which is commonly used to treat hypertension during pregnancy. This dilution of effect was expected, as PE itself often indicates early delivery, and also in this cohort was strongly associated with gestational age. However, sensitivity analysis excluding the eight subjects who had given birth before 34 gestational weeks yielded similar results to the main analyses. Only four infants were born SGA, and no effects were observed in the metabolome in association with relative birth weight, probably due to small numbers.

Quantitative PCR analysis was performed to determine whether the meconium samples contained bacterial DNA. Delay between delivery and meconium sampling resulted in a tendency towards more often positive qPCR status, but the difference was not statistically significant. See Table 2 for details. We did not identify statistically significant effects of bacterial positivity on meconium metabolites.

**Table 2:** Bacterial qPCR results according to delay in sample acquirement. Five samples were suspected to be contaminated and were excluded from this analysis, and in one sample the sampling time was not recorded

qPCR		Meconium sampled ≤ 24 h after birth *n* = 84			Meconium sampled > 24 h after birth *n* = 6			*p* value
		n	%		n	%		0.525
Positive		36	42.9		4	66.7		
Negative		25	29.8		1	16.7		
Inconclusive		23	27.4		1	16.7		

qPCR, Quantitative polymerase chain reaction

## Discussion

In this study we report differences in the meconium metabolome of infants from PE and normotensive pregnancies. Main finding is that several acylcarnitines, androsterone sulfate, three bile acids, amino acid derivatives phenylacetylglutamine, N-(phenylacetyl)glutamic acid, epsilon-(gamma-glutamyl)lysine, N-acetylhistidine and histidine betaine, as well as caffeine and paraxanthine were lower in the PE group compared to the control group. Urea and progesterone were higher in the PE group.

In our data, lower abundances of several acylcarnitines were observed in meconium samples of infants whose mothers had PE. Carnitine is both actively transported from the mother to the fetus through the placenta and synthetized by the fetus and the placenta (Oey et al., 2006). Acylcarnitines are carnitine esters produced and used in cellular energy metabolism pathways in β-oxidation of fatty acids (Dambrova et al., 2022), and an opposite association has been observed in both maternal and umbilical cord blood in previous reports on PE (Illsinger et al., 2010; Jääskeläinen et al., 2018; Thiele et al., 2004). Abnormalities in fatty acid oxidation in PE have been repeatedly reported (Branch et al., 1994; Demir et al., 2011; Uotila et al., 1998; Wakatsuki, 2000). Levels of L-carnitine and acylcarnitines in the fecal metabolome have been observed to decline during the first years of life, peaking in samples from newborns. This could be related to cessation of nutritional supply from the mother and the newborns relying more to fatty acid metabolism in a fasting-like state (Ouyang et al., 2023).

Lower abundances in bile acids sulfocholic acid, glycocholic acid and taurocholic acid were observed in the PE group. Bile acids are cholesterol derivatives involved in dietary fat digestion. Increased concentration of sulfate containing bile acids have been observed in feces of mice with nonfunctional leptin receptor, widely used to study type 2 diabetes, which may be associated with an activated pathway to remove elevated fatty acids (Walker et al., 2014). Diabetes is a well-known risk factor for PE (Bartsch et al., 2016). Bile acids are capable of influencing lipid and energy metabolism through the farnesoid X receptor and Takeda G protein-coupled receptor 5, and by inducing release of glucagon-like peptide 1 and fibroblast growth factor 19 (Huang et al., 2016; Keitel et al., 2010; Maruyama et al., 2002; Mertens et al., 2017). Therefore, the observed changes in the bile acid levels in the meconium samples from PE pregnancies could be associated with the low levels of acylcarnitines and altered lipid metabolism.

Furthermore, differences in abundances of several steroids were observed to be associated with PE. Abundance of androsterone sulfate was lower in the PE group. Sulfated steroids have historically been considered as metabolic end products as they can be easily excreted (Mueller et al., 2015), but they also act as steroid storages (Luu-The, 2013). Androsterone is a testosterone metabolite with anticonvulsant properties (Kaminski et al., 2005). However its role in PE or eclampsia has not been studied. Moreover, increased abundance of progesterone was observed in the PE group. The role of progesterone in PE is controversial, as higher maternal serum levels in PE patients have been observed (Salas et al., 2006; Zheng et al., 2016), but several studies have shown beneficial effects of progesterone on placental function, blood pressure and symptoms of PE (Liu et al., 2023; Sammour et al., 2005; You et al., 2020), and even potential for preventing PE (Melo et al., 2024).

Abundances of differing amino acids and their derivatives between the groups were all lower in the PE group in meconium samples. Contrastingly, elevated plasma levels of phenylacetylglutamine, which is metabolized from phenylacetic acid produced by gut microbes from dietary phenylalanine, have been associated with cardiovascular morbidity (Romano et al., 2023) and ischemic stroke (Yu et al., 2021), possibly acting directly on platelet aggregation (Nemet et al., 2020). Further, N-(phenylacetyl)glutamic acid is a metabolic marker of ischemic heart disease (Fromentin et al., 2022). Epsilon-(gamma-glutamyl) lysine, needed in transglutaminase activity (Griffin et al., 2002), in turn modulates cardiovascular disease by affecting angiotensin receptor signaling, platelet function, vascular structure, and inflammation (Sane, 2007). Both preexisting as well as later onset cardiovascular morbidity have consistently been associated with PE (Bartsch et al., 2016; Kattah, 2020; Seely et al., 2021).

We found lower abundances of caffeine, paraxanthine and quinic acid in the PE group compared to the control group. Increased levels of caffeine and its metabolites in maternal plasma have been observed in patients with PE compared to healthy women (Jääskeläinen et al., 2021), but caffeine intake has been controversially linked to lower risk of hypertensive pregnancy complications (Bakker et al., 2011). In another study this effect was attributed to fast caffeine metabolism rather than to caffeine consumption itself (Eichelberger et al., 2015). Quinic acid, found in coffee and many plant products, has antioxidant properties (Heikkilä et al., 2019). Increased oxidant generation has been suggested to contribute to endothelial dysfunction in PE (Watanabe et al., 2012).

Urea was observed to have higher abundances in the meconium samples of the PE group. This is in line with previous research reporting increased urea levels in preeclamptic maternal and fetal plasma, showing that disturbances in urea cycle are linked to PE (Jääskeläinen et al., 2018, 2021).

Previous studies indicate that meconium metabolite composition is strongly driven by the time post-partum (Bekhti et al., 2022), reflecting the start of the feeding and bacterial colonization of the neonatal gut (Bittinger et al., 2020). We collected the samples immediately after the first meconium appearance and used a qPCR approach for determining whether they contained bacterial DNA over negative controls. While slightly less than half of the samples collected ≤ 24 h after birth were positive, bacterial positivity was not reflected in the meconium metabolome. We did appropriately control for contaminants during the laboratory procedures, but it should be noted that the meconium microbiota, when detected, typically consists of somewhat stochastic microorganisms acquired *ex utero* i.e. from the skin or maternal vaginal or gut microbiota (Kennedy et al., 2023). Not all of these transient, non-colonizing bacteria are expected to be metabolically active in the neonatal gut, possibly explaining the low effect of bacterial presence on meconium metabolome at the time of first passing of the meconium. Some of the differentially abundant metabolites may however be linked to the maternal microbiota, as they were less abundant in fetuses of germ-free mice (Pessa-Morikawa et al., 2022), or are known to be produced by bacteria.

We excluded women with pregestational or medically treated gestational diabetes to reduce confounding, but included milder phenotypes to avoid selection bias, as gestational diabetes (GDM) is associated with increased PE risk (The HAPO Study Cooperative Research Group, 2008). GDM has also been shown to have an effect on meconium metabolome (Chen et al., 2021; Peng et al., 2015). Diet-controlled GDM did not affect the meconium metabolome in our study, which may be due to including only patients with a mild disorder.

A strength of our study is the homogenous study population, representing pregnancies of women with similar genetic background living in the same region. Further, using only one study center permitted standardized sampling of meconium and good adherence to the study protocol. Regarding method of metabolomics analyses, we chose to use non-targeted LC-MS with four different analytical modes to get a wide and relatively unbiased measure of the metabolite profile of meconium samples.

The weaknesses of our study include the failure to match cases and controls regarding gestational age. However, PE very often leads to expedited delivery, and lower gestational age at delivery compared to general obstetric population could be seen as part of the disorder. Strictly matching controls by gestational age, especially preterm, would lead to further confounding due to other complications causing the earlier delivery (such as infections). Main results persisted when early or moderately preterm births (< 34 gestational weeks) were excluded, which supports that our findings are associated with PE and not prematurity. Another weakness is the size of the cohort, as many results were of borderline statistical significance. Our population consists of Finnish women, and further research is needed to validate results in different ethnicities. Further, LC-MS observes a wide range of hydrophilic metabolites and polar lipids, but does not include all metabolites, e.g. non-polar lipids like triglycerides.

To our knowledge, this is the first study to describe alterations in the meconium metabolome in association with PE, which also complicates the interpretation of the findings. The differing abundances of several metabolites linked to fatty acid, steroid and bile acid, urea and amino acid metabolism confirm alterations in the interaction between the fetoplacental unit and the mother in PE, but whether they are a cause of an effect of the disorder remains to be further investigated. The pathways leading to differences in meconium metabolome need to be further elucidated: are differing abundances due to excess or shortage of certain compounds or instead due to altered excretion of them? Our study described the first stool of the newborn, formed in intrauterine period but passed after contact with the extrauterine environment. Persisting alterations in metabolic pathways later in life could explain the increased morbidity in offspring after a PE pregnancy, but further research is needed to evaluate how environmental and other factors modify these associations.

## Electronic supplementary material

Below is the link to the electronic supplementary material.

Supplementary Material 1

Supplementary Material 2

## Data Availability

The authors confirm that access restrictions apply to the data. The GDPR legislation requires us to protect the identity of participants, and the raw data cannot be publicly shared.
