# Supplementary material for: Metabolic profiles of meconium in preeclamptic and normotensive pregnancies
Source: Metabolomics. 2025 Jan 25;21(1):21. doi: 10.1007/s11306-025-02224-4 (PMC11762436; doi:10.1007/s11306-025-02224-4)
Supplement: Supplementary file 1 — Supplementary Material 1 [file 11306_2025_2224_MOESM1_ESM.docx]

**Corresponding author: Elli Toivonen, e-mail: elli.toivonen@tuni.fi**

This supplementary file is divided into two subsections: 1) supplementary methods and 2) supplementary data.

1. **Supplementary methods**
   1. *Reagents and solvents*

- Acetonitrile, ACN (HiPerSolv CHROMANORM, VWR Chemicals, Fontenay-sous-Bois, France, Cat.No. 83640.320)
- Methanol, MeOH (CHROMASOLV™ LC–MS Ultra, Riedel-de Haën™, Honeywell, Seelze, Germany, Cat.No. 14262-2L)
- Formic acid (HiPerSolv CHROMANORM, VWR Chemicals, Leuven, Belgium, Cat.No. 84865.180)
- Ammonium formate (HiPerSolv CHROMANORM, VWR Chemicals, Leuven, Belgium, Cat.No. 84884.180)
- Ultra-pure water, ultrapure (Type 1), Direct-Q®, Merck KGaA, Darmstadt, Germany
  1. *LC-MS analytical parameters*

The following conditions were used for the reversed-phase (RP) chromatography: Column oven temperature 50 °C, flow rate 0.4 mL/min, gradient elution with water (eluent A) and methanol (eluent B) both containing 0.1% (v/v) of formic acid. The gradient profile for the RP separations was as follows: 0–10 min: 2 → 100% B; 10–14.5 min: 100% B; 14.5–14.51 min: 100 → 2% B; 14.51–16.5 min: 2% B. Needle wash was performed with 50% MeOH. The injection volume was set at 2 μL and the sample tray at 10 °C.

The following conditions were used for the hydrophilic interaction chromatography (HILIC): Column oven temperature 45 °C, flow rate 0.6 mL/min, gradient elution with 50% v/v ACN in water (eluent A) and 90% v/v ACN in water (eluent B), both containing 20 mM ammonium formate. The gradient profile for HILIC separations: 0–2.5 min: 100% B, 2.5–10 min: 100% B → 0% B; 10–10.01 min: 0% B → 100% B; 10.01–12.5 min:100% B. Needle wash with 50% ACN. The injection volume was set at 2 μL and the sample tray at 10 °C.

To operate at high mass accuracy, the MS was calibrated daily, and the continuous mass axis calibration was used by monitoring two reference ions from an infusion solution throughout the analytical runs. The reference ions in ESI+ mode: m/z 121.050873 and m/z 922.009798, and reference ions in ESI− mode m/z 119.03632 and m/z 966.000725. These reference ions are coming from the compounds in the infusion solution where m/z 121 and 119 are purine, and m/z 922 and 966 are HP-0921 (Hexakis (1H,1H,3H-tetrafluoropropoxy) phosphazine).

The following conditions were used for the Dual AJS ESI source: drying gas temperature 325 °C and flow 10 L/min, sheath gas temperature 350 °C with a flow of 11 L/min, nebulizer pressure 45 psi, capillary voltage 3500 V, nozzle voltage 1000 V, fragmentor voltage 100 V and skimmer 45 V. Nitrogen was used as the instrument gas.

For data acquisition, a 10 GHz extended dynamic range mode was used in both ESI+ and ESI– ionization modes from m/z 50 to 1600. The data was collected in the centroid mode at an acquisition rate of 1.67 spectra/s (i.e., 600 ms/spectrum) with an abundance threshold of 150. For automatic data-dependent MS/MS analyses, the precursor isolation width was set to 1.3 Da. From every precursor scan cycle, the four most abundant ions were selected for fragmentation. These ions were excluded after two product ion spectra and released again for fragmentation after a 0.25-min hold. Product ion scan time was based on precursor ion intensity, ending at 25,000 counts for RP modes and 20,000 for HILIC modes, or after 300 ms. Collision-induced dissociation voltages 10, 20, and 40 V were used for MSMS acquisition.

- 1. *Sequence setup*

Two blank samples were injected at the beginning of each analytical run, followed by at least 10 injections of pooled quality control (QC) sample to stabilize the signals. QCs were also injected after 12 study samples. MSMS data was acquired from QC samples and two blank injections at the end of the chromatography.

- 1. *Peak picking and alignment*

The peak picking and alignment was performed in MS-DIAL for each mode separately. For the feature extraction, we used MS1 and MS2 tolerance 0.01 Da and 0.025 Da, respectively, minimum peak height 3000 amplitude, mass slice width 0.1 Da, smoothing level 3 scans, and minimum peak width 5 scans. For the initial annotation, we used a database file compiled from our in-house spectral database (for the level 1 identifications), public spectral libraries available from the MS-DIAL repository, and MassBank of North America (MoNA). We considered [M+H]^+^, [M+NH_4_]^+^, [M+Na]^+^, [M+CH_3_OH+H]^+^, [M+K]^+^, [M+H−H_2_O]^+^, [M+H−2H_2_O]^+^, [2M+H]^+^, and [M−NH_4_+H]^+^ as adducts in the positive mode and [M−H]^−^, [M−H_2_O]^−^, [M+Cl]^−^, [M+FA−H]^−^, and [2M−H]^−^ in the negative mode. For feature alignment, we used retention time tolerance 0.2 min and MS1 tolerance 0.015 Da, no peak count filters, a sample-maximum-to-blank-average ratio of minimum 5, and gap filling by compulsion.

- 1. *Data preprocessing*

All the analytical modes were preprocessed separately and were combined before statistical analysis. Molecular features were considered of high quality if they met all the following quality metrics, as described in more detail by Broadhurst *et al.* 2018 and Klåvus *et al.* 2020: low number of missing values (present in more than 70% of the QC samples, present in at least 50% of samples in at least one study group) and RSD* below 20%, D-ratio* below 10%. In addition, if either RSD* or D-ratio* was above the threshold, the features were still considered good quality if their classic RSD, RSD* and basic D-ratio were all below 10%.

Drift correction was performed using method described in Klåvus *et al.* 2020. Firstly, abundance values were log-transformed to conform better with model assumptions. Secondly, drift was modelled by fitting a smoothed cubic spline to QC samples. Finally, all samples were corrected with the following formula:

$$x_{corrected}\left( i \right)=\exp\left( x_{original}\left( i \right)+ mean\left( x_{QC} \right)- f_{drift}\left( i \right) \right)$$

where the exponential function reverses prior log-transformation.

- 1. *Compound identification*

We used the automatic annotation provided by MS-DIAL and the database file, consisting of our in-house library and public spectral libraries available from the MS-DIAL repository and MassBank of North America (MoNA). The tolerances for MS1 and MS2 peaks were 0.01 and 0.05 Da, respectively. The retention time match for the level 1 identifications was considered individually during the manual inspection as long as the difference did not exceed 0.5 min.

- 1. *Evaluating possible modulation/origin from gut commensals*

The metabolomic data acquired in these analyses were aligned to a previously published dataset of metabolites modulated by gut microbiota using the latter as a reference, by authors MN and AM. The reference LC-MS data were obtained using the same LC system, the same elution, and mass-spectrometry parameters as in the present study (see above and Pessa-Morikawa et al., 2022). At the first stage, both LC-MS datasets were separately processed in MS-Dial with parameters listed above (peak-picking and alignment). The resulting spectra from the alignment of the reference dataset were exported as an msp-file and used at the second stage as a reference database for compounds identification (the tolerance for MS1 and MS2 signals were 0.01 and 0.025 Da, respectively; for retention time 0.3 min; minimum of three matched spectra per molecular feature). All matches with the compounds previously revealed as microbially modulated were curated manually based on MS2 data, retention time, and m/z values, engaging Sirius v. 5.8.6 (Dührkop et al., 2019) as an additional tool for structure comparison and interpretation.

- 1. *Nested PCR approach for testing bacterial positivity of meconium samples*

Meconium samples were subjected for DNA extraction using ca. 200 mg of sample and mechanical cell lysis as described in detail by Dubois et al. (Dubois et al., 2024). The DNA was quantified with fluorometry (Quant-IT Quant-iT™ dsDNA Assay Kits, high sensitivity (HS), Thermo Fisher Scientific). The median DNA concentration was 0,1 ng/μl. We first tested a qPCR approach without a probe (Jokela et al., 2022), but human DNA extracted from blood used as a negative control gave signal that could not be reliably differentiated from the samples in melting curve analysis. The same assay with a probe (Husso et al., 2020) was highly specific but less sensitive, and we wound have ran out of samples to run them in triplicates.

Hence, due to the very low DNA concentrations, a nested PCR approach was applied to categorize the meconium samples as negative or positive in terms of bacterial DNA. Nested PCR consists of two successive PCRs, in our case the first PCR targeting the full-length 16S RNA gene found in all bacteria, and the second PCR a shorter fragment of the same gene. Nested PCR has been used to study low bacterial biomass samples that have high host DNA content as it helps to increase assay sensitivity without compromising the specificity if controlled appropriately by negative controls (Brun et al., 2020; Smolejová et al., 2021).

We first enriched bacterial DNA with primers targeting the full-length 16S rRNA gene (ca. 1500

Bp, primers F:5’-GAGAGTTTGATYCTGGCTCAG-3’ and R:5’-

AAGGAGGTGATCCARCCGCA -3’ (Wang et al., 2002). Non-template negative controls were included in the DNA extraction and both PCR steps. Human DNA extracted from blood was used as another negative control. Positive control consisted of genomic DNA purified from a pure culture of *Bifidobacterium bifidum* in 1x10^3^ copies, representing the detection limit of the assay. The amount of meconium DNA in the reaction was 5 μl and the mastermix was as follows: 4.25 μl water; 1.25 µl 10 μM primers (reverse 5'-AAGGAGGTGATCCARCGCA-3'; forward 5'-GAGAGTTTGATYCTGGCTCAG-3'); 0.75 μl DMSO and 12.5 μl Phusion High-Fidelity PCR Master Mix with HF Buffer. The PCR run was performed using a Bio-Rad CFX96 Touch System C1000 Touch Thermal Cycler and the program was as follows: initial denaturation at 98 C for 2 minutes followed by 15 cycles of 1) denaturation at 94° C for 20 seconds, 2) annealing at 55 C for 20 seconds, 3) extension at 72° C for 60 seconds and then final extension at 72 C for 5 minutes.

The PCR-products were then cleaned using ExoSAP-IT PCR Product Cleanup (Applied Biosystems by Thermo Fisher Scientific) according to the manufacturer's instructions.

Next, the PCR-products were subjected to quantitative PCR (qPCR) using widely used universal bacterial 16S rRNA gene qPCR primers (Nadkarni et al., 2002). The amount of PCR product in the reaction was 1 μl and the mastermix was as follows: 18.8 μl water; 0.1 μl 50 μM primers (reverse 5´-GGACTACCAGGGTATCTAATCCTGTT-3´; forward 5´-TCCTACGGGAGGCAGCAGT-3´) and 5 μl 5x HOT FIREPol^®^ EvaGreen^®^ qPCR Mix Plus (no ROX; Solis BioDyne, Tartu, Estonia). The PCR run was performed using a Bio-Rad CFX96 Touch System C1000 Touch Thermal Cycler and the program was as follows: DNA-denaturation step at 95° C for 15 minutes, followed by 40 cycles of 1) denaturation at 95° C for 15 seconds, 2) annealing at 50 C for 20 seconds, 3) extension at 72° C for 30 seconds and 4) an incubation step at 82°C to detect the fluorescent data. A melting curve analysis was carried out to ensure the specificity of the amplification products.

All qPCR assays were performed in triplicate and negative and positive controls were included in all steps along with the samples. Precautions were taken to ensure that the data from each triplicate fell within 0.5 threshold cycle (Ct) and outliers were removed before calculating average Ct of each sample. Melting curves and non-template control from qPCR were used to assess run reliability. The Ct values and melting curves were used to determine bacterial positivity of the samples. Samples were considered positive when their quantification cycle (Cq)-value was lower than that of negative controls on the same plate, and melting curve analysis indicated a specific product similar to the positive control. If only one of these criteria were met the samples were coded inconclusive, and samples with the same or higher Cq than negative controls were coded as negative.

Brun, A., Rangé, H., Prouvost, B., Mazighi, M., Kapila, Y., Bouchard, P., & Michel, J.-B. (2020). Innovative application of nested PCR for detection of *Porphyromonas gingivalis* in human highly calcified atherothrombotic plaques. *Journal of Oral Microbiology*, *12*(1), 1742523. https://doi.org/10.1080/20002297.2020.1742523

Dubois, L., Valles-Colomer, M., Ponsero, A., Helve, O., Andersson, S., Kolho, K.-L., Asnicar, F., Korpela, K., Salonen, A., Segata, N., & De Vos, W. M. (2024). Paternal and induced gut microbiota seeding complement mother-to-infant transmission. *Cell Host & Microbe*, *32*(6), 1011-1024.e4. https://doi.org/10.1016/j.chom.2024.05.004

Dührkop, K., Fleischauer, M., Ludwig, M., Aksenov, A. A., Melnik, A. V., Meusel, M., Dorrestein, P. C., Rousu, J., & Böcker, S. (2019). SIRIUS 4: A rapid tool for turning tandem mass spectra into metabolite structure information. *Nature Methods*, *16*(4), 299–302. https://doi.org/10.1038/s41592-019-0344-8

Husso, A., Jalanka, J., Alipour, M. J., Huhti, P., Kareskoski, M., Pessa-Morikawa, T., Iivanainen, A., & Niku, M. (2020). The composition of the perinatal intestinal microbiota in horse. *Scientific Reports*, *10*(1), 441. https://doi.org/10.1038/s41598-019-57003-8

Jokela, R., Korpela, K., Jian, C., Dikareva, E., Nikkonen, A., Saisto, T., Skogberg, K., De Vos, W. M., Kolho, K.-L., & Salonen, A. (2022). Quantitative insights into effects of intrapartum antibiotics and birth mode on infant gut microbiota in relation to well-being during the first year of life. *Gut Microbes*, *14*(1), 2095775. https://doi.org/10.1080/19490976.2022.2095775

Nadkarni, M. A., Martin, F. E., Jacques, N. A., & Hunter, N. (2002). Determination of bacterial load by real-time PCR using a broad-range (universal) probe and primers set. *Microbiology*, *148*(1), 257–266. https://doi.org/10.1099/00221287-148-1-257

Pessa-Morikawa, T., Husso, A., Kärkkäinen, O., Koistinen, V., Hanhineva, K., Iivanainen, A., & Niku, M. (2022). Maternal microbiota-derived metabolic profile in fetal murine intestine, brain and placenta. *BMC Microbiology*, *22*(1), 46. https://doi.org/10.1186/s12866-022-02457-6

Smolejová, M., Cihová, I., & Sulo, P. (2021). Reliable and Sensitive Nested PCR for the Detection of Chlamydia in Sputum. *Microorganisms*, *9*(5), 935. https://doi.org/10.3390/microorganisms9050935

Wang, R. F., Kim, S.-J., Robertson, L. H., & Cerniglia, C. E. (2002). Development of a membrane-array method for the detection of human intestinal bacteria in fecal samples. *Molecular and Cellular Probes*, *16*(5), 341–350. https://doi.org/10.1006/mcpr.2002.0432

1. **Supplementary data**
   1. *Outlier removal*

While the PCA did not show outlier samples, two samples were removed from the statistical analysis due to their very low overall abundance values (Figure 1), suggesting an issue during the analysis, such as sample injection.


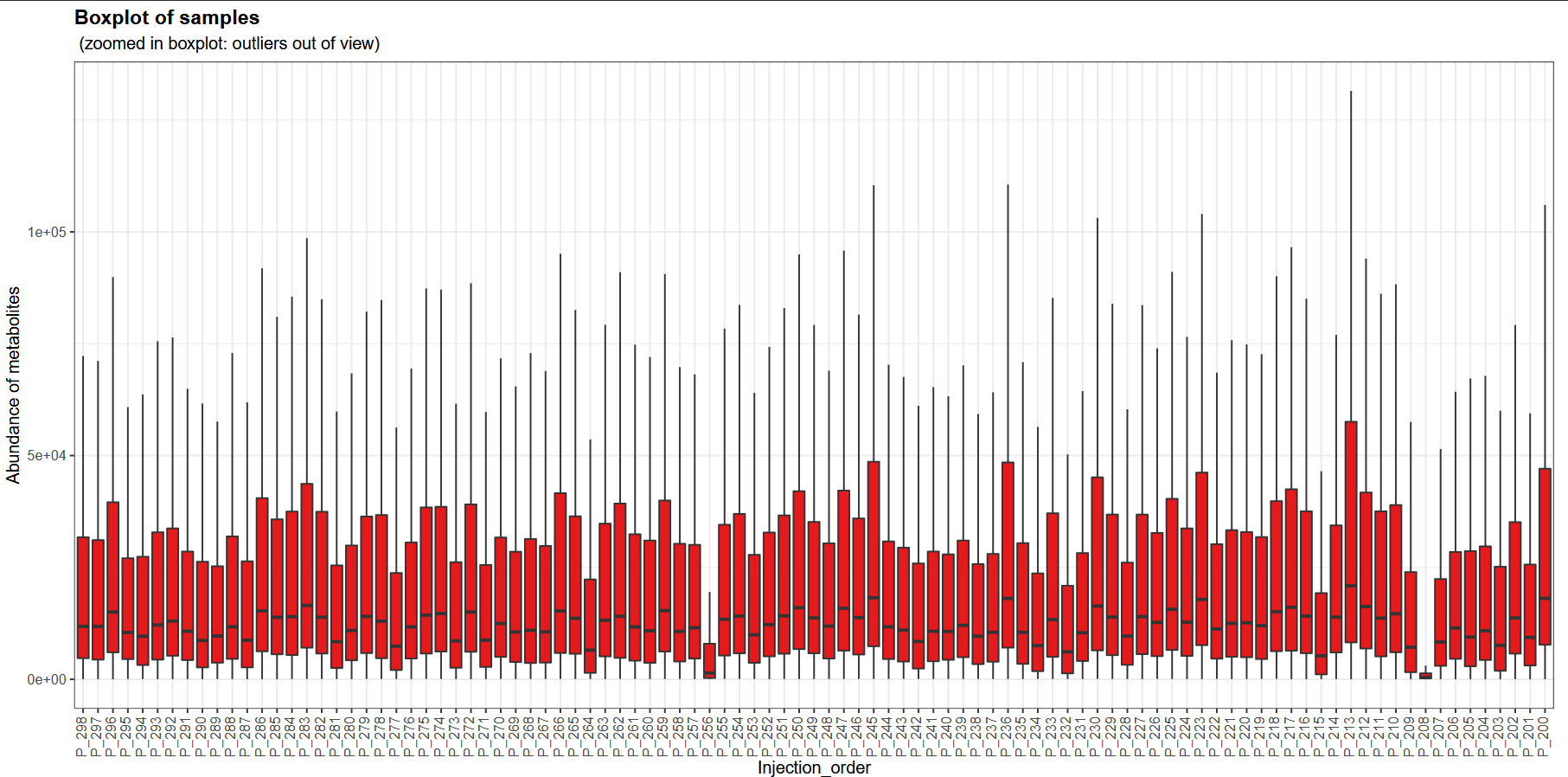


Figure 1. Boxplots of the detected signal abundances across all samples. Samples P_256 and P_208 showed a very low relative abundance compared to the other samples.
